# Buyang Huanwu Decoction attenuates H_2_O_2_-induced apoptosis by inhibiting reactive oxygen species-mediated mitochondrial dysfunction pathway in human umbilical vein endothelial cells

**DOI:** 10.1186/s12906-016-1152-7

**Published:** 2016-05-31

**Authors:** Jian Shen, Yu Zhu, Kaiyuan Huang, Hao Jiang, Chengzhang Shi, Xiaoxing Xiong, Renya Zhan, Jianwei Pan

**Affiliations:** Department of Neurosurgery, First Affiliated Hospital, School of Medicine, Zhejiang University, No. 79 Qingchun Road, Hangzhou, Zhejiang People’s Republic of China; Department of Anesthesiology, First Affiliated Hospital, School of Medicine, Zhejiang University, Hangzhou, Zhejiang Province People’s Republic of China

**Keywords:** Buyang Huanwu Decoction, Reactive oxygen species, Apoptosis, Ritochondria, Cerebral ischeima

## Abstract

**Background:**

Apoptosis of endothelial cells caused by reactive oxygen species plays an important role in ischemia/reperfusion injury after cerebral infarction. Buyang Huanwu Decoction (BYHWD) has been used to treat stroke and stroke-induced disability, however, the mechanism for this treatment remains unknown. In this study, we investigated whether BYHWD can protect human umbilical vein endothelial cells (HUVECs) from H_2_O_2_-induced apoptosis and explored the underlying mechanisms.

**Methods:**

To investigate the effect of BYHWD on the apoptosis of HUVECs, we established a H_2_O_2_-induced oxidative stress model and detected apoptosis by Hoechst 33342 and propidium iodide staining. JC-1 and DCFH-DA assays,western blotting and electron microscopy were used to examine the mechanism of BYHWD on apoptosis.

**Results:**

Pretreatment with BYHWD significantly inhibited H_2_O_2_-induced apoptosis and protein caspase-3 expression in a concentration-dependent manner. In addition, BYHWD reduced reactive oxygen species production and promoted endogenous antioxidant defenses. Furthermore, loss of mitochondrial membrane potential and structural disruption of mitochondria were both rescued by BYHWD.

**Conclusions:**

BYHWD protects HUVECs from H_2_O_2_-induced apoptosis by inhibiting oxidative stress damage and mitochondrial dysfunction. These findings indicate that BYHWD is a promising treatment for cerebral ischemia diseases.

## Background

Stroke is the second leading cause of death and a major cause of disability worldwide. About 85–90 % of strokes are caused by ischemia (resulting from arterial occlusion) [1]. Excessive production of reactive oxygen species (ROS) such as H_2_O_2_, superoxide radicals, and hydroxyl radicals has been observed during cerebral ischemia/reperfusion (I/R) [2, 3]. This elevated ROS production alters mitochondrial permeability, which reduces mitochondrial membrane potentials (MMP), causing the release of Cyt-c. This activates caspase signaling pathways, which are important mediators of apoptosis [4–6]. Therefore, excessive ROS levels induce mitochondrial dysfunction, which promotes ROS-mediated apoptosis [7]. Preliminary studies have shown that ROS-induced apoptosis of vascular endothelial cells aggravates secondary brain injury after cerebral infarction [8, 9]. Protecting vascular endothelial cells against ROS-induced apoptosis may therefore have a therapeutic benefit in cerebrovascular diseases.

Numerous clinical trials have demonstrated that BYHWD improves the outcomes of ischemic stroke [10]. Recent studies have reported neuroprotective effects of BYHWD against cerebral I/R injury in animal experiments [11, 12]. BYHWD may also inhibit the apoptosis of nerve cells caused by I/R injury [13]. However, the mechanism behind the anti-apoptotic activity of BYHWD in endothelial cells is not well defined. Our previous findings have indicated that BYHWD is involved in angiogenesis by enhancing angiopoietin-1 expression after focal cerebral ischemia in rats [14]. In this study, we investigated the protective effects of BYHWD on H_2_O_2_-induced apoptosis in human umbilical vein endothelial cells (HUVECs) and explored the underlying mechanisms.

## Methods

### Composition and preparation of BYHWD

BYHWD was prepared with the following components: Radix Astragali (Shanxi, China), Radix Angelicae Sinensis (Gansu, China), Radix Paeoniae Rubra (Sichuan, China), Rhizoma Ligustici Chuanxiong (Sichuan, China), Semen Persicae (Sichuan, China), Flos Carthami (Henan, China), and Pheretima (Guangdong, China). These components were mixed at a ratio of 120:10:10:10:10:10:4.5 (dry weight) [13]. All ingredients were purchased from the East China Pharmaceutical Group Co., Ltd., Zhejiang Province, China, and deposited at the Department of Pharmacy, Zhejiang University after verification by Professor Dong at the same institute. The decoction was made by boiling the mixture in ten times the amount of distilled water at 100 °C for 30 min. Then, the drug solution was poured out for use and the residue boiled two more times. The total drug solution for three times was vacuum-cooled and dried to a powder, which was dissolved in distilled water at a final concentration of 2.0 g/ml (equivalent to the dry weight of the raw materials).

### Qualitative and quantitative analysis of active ingredients

Based on the theories of traditional Chinese medicine, a herbal formulation contains more than one Chinese herb. According to the literature, the effective components of BYHWD are astragaloside IV, paeoniflorin, amygdalin, and tetramethylpyrazine. These active ingredients were quality controlled by high-performance liquid chromatography (HPLC) in our study [15]. Standard chemicals including astragaloside IV, paeoniflorin, amygdalin, and tetramethylpyrazine were purchased from the Biological Products Analysis Bureau at the Ministry of Public Health of China. Briefly, HPLC profiling was performed using an Agilent 1100 series equipped with a quaternary solvent delivery system, auto-sampler, and a photodiode array (PDA) detector (Waters Breeze, USA). Separation was performed on a Cosmosil ARII column (250 mm × 4.6 mm, 5 μm; temperature: 35 °C; flowrate: 1 ml/min; injection volume: 10 μL). The mobile phase used astragaloside IV, acetonitrile/water (33/67, v:v), paeoniflorin, amygdalin, tetramethylpyrazine, and a methanol/water (33/67, v:v) solution. The linear gradient elution was optimized for BYHWD as follows: 2–2 % B (0–5 min), 2–30 % B (5–50 min), 30–60 % B (50–70 min), with a 15-min re-equilibration of the gradient elution.

### Cell culture

HUVECs were obtained from ATCC (Rockville, MD, USA) and maintained in Dulbecco’s modified Eagle’s Medium (DMEM) (Hangzhou Sijiqing Biological Engineering Materials Co., Ltd., China) supplemented with heat-inactivated 10 % fetal bovine serum (FBS) (Hangzhou Sijiqing Biological Engineering Materials Co., Ltd., China), 100 U/ml penicillin, and 100 U/ml streptomycin in a humidified atmosphere of 5 % CO_2_ at 37 °C. Cells were used at passage 4–6 in all experiments.

### MTT assay

An MTT assay was used to estimate cell viability. Briefly, HUVECs were seeded into 96-well plates (BD Falcon, USA), at a density of 2 × 10^3^ cells/well in DMEM supplemented with 10 % FBS. One day after plating, the cells were washed and incubated in serum-free medium for 12 h. The cells were then washed again. To explore the cytotoxicity of BYHWD on HUVECs, cells were incubated with various concentrations of BYHWD (5–45 mg/ml) for 24 h. To explore the effects of BYHWD on H_2_O_2_-induced cytotoxicity, cells were pretreated for 6 h with culture medium containing different concentrations of BYHWD (5, 15, and 30 mg/ml). The cells were then exposed to H_2_O_2_ (320 μmol/l) diluted in culture medium for 6 h. After H_2_O_2_ exposure, the culture supernatant was removed and cells were washed with PBS before incubating in 150 μl MTT solution (5 mg/ml) in culture medium at 37 °C for 3 h. After removal of the MTT solution, the colored formazan crystals were dissolved in 100 μl DMSO. Plates were gently shaken for 10 min before measuring the optical density at 490 nm using an enzyme-labeled instrument (Biotek ELX 800/FLX800).

### Hoechst 33342 and propidium iodide (PI) staining

Apoptotic HUVECs were identified by Hoechst 33342 and PI (Hangzhou Baoke Biological Technology Co., Ltd., China) staining. Briefly, HUVECs were seeded in a 6-well plate (BD Falcon, USA) at a density of 1 × 10^4^ cells/well in DMEM supplemented with 10 % FBS. One day after plating, the cells were washed and incubated in serum-free medium for 12 h. The cells were then washed again and pretreated for 6 h with different concentrations of BYHWD (5, 15, and 30 mg/ml). The cells were then exposed to H_2_O_2_ (320 μmol/l) for 6 h before double-staining with Hoechst 33342 and then PI for 10 min each at 37 °C according to the manufacturer’s instructions. Cells were observed under a fluorescence microscope (Olympus, Japan). Hoechst 33342 was excited at 361 nm and emitted blue fluorescence at 460 nm. PI was excited at 480 nm and red fluorescence was detected at 610 nm. Data were analyzed using Image Pro plus software (Media Cybernetics). Data were expressed as the percentage of apoptotic cells.

### Western blotting

After incubating as described above, cells were seeded into 6-well plates. Cells were lysed in 200 μl RIPA lysis buffer (Hangzhou Baoke Biological Engineering Materials Co., Ltd., China), centrifuged at 20,000 g for 5 min at 4 °C, and the supernatant was collected. Nuclear extracts were collected according to the protocol of the nuclear extraction kit (Hangzhou Baoke Biological Engineering Materials Co., Ltd., China). Protein extracts were quantified using a BCA protein assay kit (Hangzhou Baoke Biological Engineering Materials Co., Ltd., China). Equal amounts of proteins (50 μg per lane) were separated on a 10 % SDS-polyacrylamide gel by electrophoresis and transferred onto a polyvinylidene difluoride membrane (Millipore, Milford, MA, USA). Membranes were blocked with 5 % fat-free milk at room temperature for 2 h and incubated with primary antibodies against active caspase-3 (Abcam, USA, 1:1000) or glyceraldehyde-3-phosphate dehydrogenase (GAPDH) (Santa Cruz, CA, USA, 1:1000) at 4 °C overnight. After primary antibody incubation, membranes were washed three times in TBST then incubated in secondary antibody solutions for 2 h at room temperature. After washing, the signals were visualized using enhanced chemiluminescence and X-ray film (Kodak, USA). Protein levels were quantified by densitometry. Data were expressed as the ratio of active caspase-3 to GAPDH.

### Measurement of intracellular ROS

Measurement of intracellular ROS was based on ROS-mediated conversion of non-fluorescent 2,7-DCFH-DA into fluorescent DCFH. Briefly, HUVECs were seeded into a 6-well plate (BD Falcon, USA) at a density of 1 × 10^4^ cells/well in DMEM supplemented with 10 % FBS. One day after plating, the cells were washed and incubated in serum-free medium for 12 h. The cells were washed and pretreated with BYHWD (30 mg/ml) for 6 h. The cells were then exposed to H_2_O_2_ (320 μmol/l) for 6 h before incubating in 2,7-DCFH-DA (Sigma, USA, 5 μM) in PBS for 30 min. Cells were washed three times with PBS then the DCFH fluorescence from each well was excited at 485 nm and the emission was measured at 520 nm by fluorescence microscopy (Olympus, Japan). For spectrofluorophotometry analysis, the cells were collected and analyzed. The fluorescence of DCFH was detected using a spectrofluorophotometer (Amersham Biosciences, USA). Data were expressed relative to the mean sham value.

### Measurement of superoxide dismutase (SOD) and malondialdehyde (MDA) levels

The levels of SOD and MDA were determined using commercially available kits (Nanjing Jiancheng Biological Engineering Materials Co., Ltd., China). Briefly, after incubating as described above, cells were lysed and centrifuged at 12,000 g for 10 min at 4 °C, to collect the supernatant. The SOD and MDA levels in the supernatant were measured according to the kit protocol and analyzed on a spectrophotometer,The absorbance was read at 550 nm (SOD) and 450 nm (MDA). The levels were calculated using a standard calibration curve and expressed in U/mg (SOD) and nmol/ml (MDA). Data were expressed as relative to the mean sham value.

### Transmission electron microscopy

Electron microscopy was performed to examine the morphology of the mitochondria in HUVECs. Briefly, after incubating as described above, cells were collected and prefixed with 2.5 % w/v glutaraldehyde for 2 h, rinsed three times in 0.1 mol/l PBS (pH 7.4), and post-fixed for 2 h in 1 % w/v osmium tetroxide at −4 °C. The fixed cells were dehydrated through an ethanol series then completely dehydrated in absolute ethanol. Cells were detached using propylene oxide and infiltrated with Spurr low-viscosity embedding medium (Wuhan Boshide Biological Technology Co., Wuhan, China). Sections were cut using an ultramicrotome with a diamond knife, and stained with uranyl acetate and lead citrate. Cells were observed using a transmission electron microscope (JEM-1230, Olympus Corporation, Tokyo, Japan).

### Measurement of MMP

MPP was assessed using the lipophilic cationic probe JC-1, which is a sensitive fluorescent dye (Invitrogen, USA). Briefly, after incubating as described above, cells were incubated with 5 mM JC-1 dye (Molecular Probes) at 37 °C for 15 min. Cells were then washed three times with PBS and immediately analyzed with a fluorescence microscope (Olympus, Japan). Red emission indicated membrane potential-dependent JC-1 aggregates in the mitochondria. Green fluorescence indicated the monomeric form of JC-1 entering the cytoplasm after mitochondrial membrane depolarization. The ratio of red to green fluorescence intensity was measured and data were expressed as relative to the mean sham value.

### Statistical analysis

All data were expressed as the mean ± standard deviation (SD). Statistical analysis was performed using the Student’s *t*-test and ANOVA. Significance was accepted at *P*<0.05. SPSS 19.0 software was used for statistical analysis.

## Results

### Qualitative and quantitative analysis of BYHWD components

**Fig. 1:**
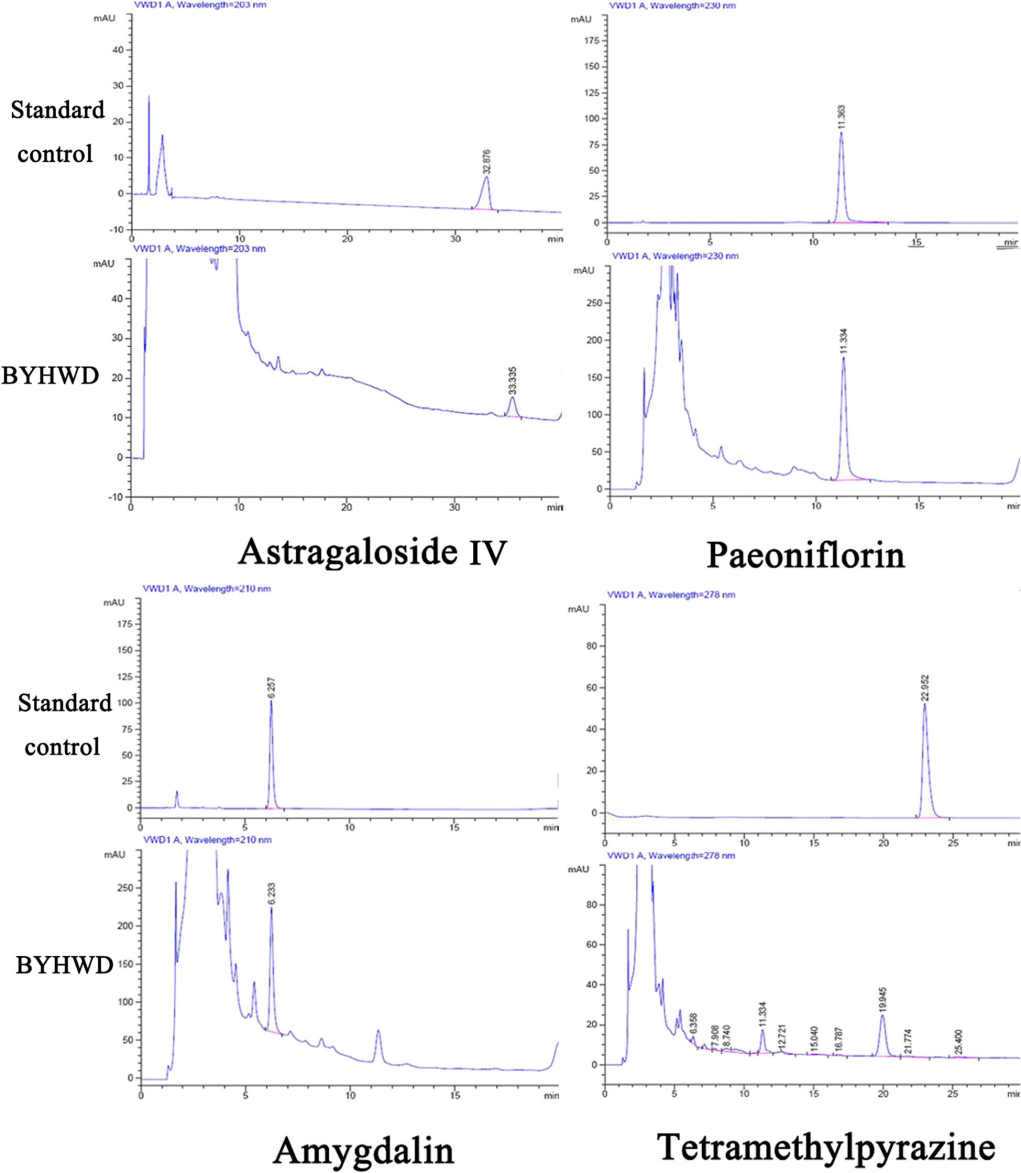
Representative HPLC chromatograms of active BYHWD components and standard controls. BYHWD contains astragaloside IV, paeoniflorin, and amygdalin, but not tetramethylpyrazine. Summit retention times of astragaloside IV, paeoniflorin, and amygdalin were 35 min, 11.5 min and 6 min, respectively

To identify BYHWD extracts containing active ingredients, quality control was performed by HPLC. BYHWD contained astragaloside IV, paeoniflorin, and amygdalin, but not tetramethylpyrazine (Fig. 1). The summit retention time of astragaloside IV, paeoniflorin, and amygdalin was approximately 35 min, 11.5 min, and 6 min, respectively. The content of astragaloside IV, paeoniflorin, and amygdalin was 0.58, 1.63, and 1.82 mg/g, respectively.

### Cytoactivity and cytotoxicity of BYHWD in HUVECs

**Fig. 2:**
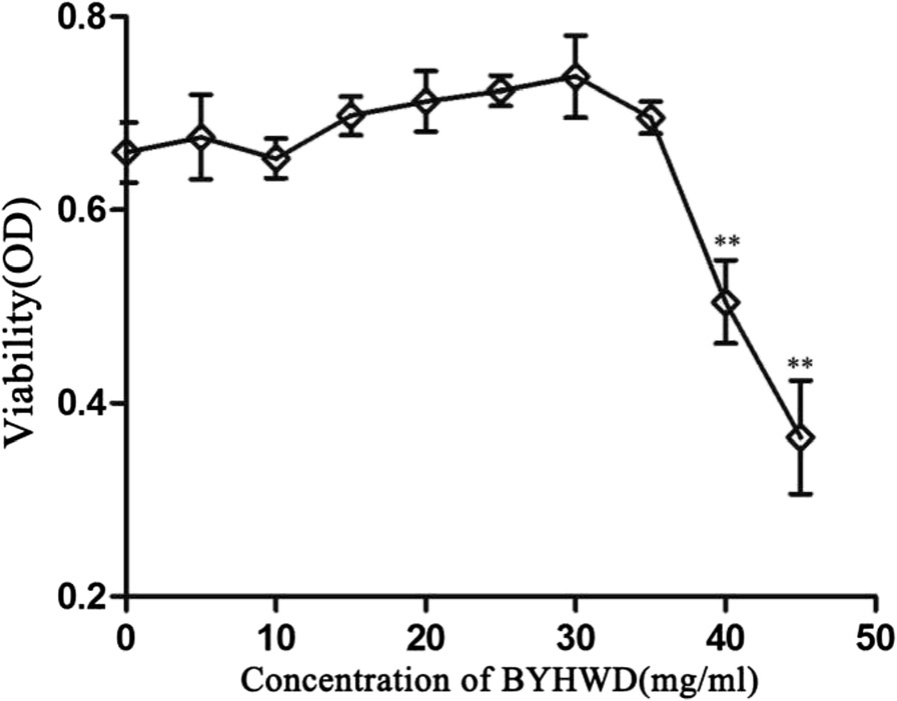
Cytoactivity and cytotoxicity of HUVECs treated with various concentrations of BYHWD. HUVEC viability after treatment with different concentrations of BYHWD (5–45 mg/ml) for 24 h. Values are shown as means ± SD (*n* = 6).^**^ *P* < 0.01, compared to sham group

To determine the optimal experimental concentration of BYHWD, we measured the viability of HUVECs after treatment with various concentrations of BYHWD by MTT assay. Cytoactivity was not significantly increased by BYHWD at concentrations of 5–30 mg/ml, but cytotoxicity increased in a dose-dependent manner at concentrations of 35–45 mg/ml (Fig. 2). To investigate whether effects of BYHWD were dose-dependent, we selected low (5 mg/ml), medium (15 mg/ml) and high (30 mg/ml) doses for experiments.

### BYHWD inhibits H_2_O_2_-induced cytotoxicity in HUVECs

**Fig. 3:**
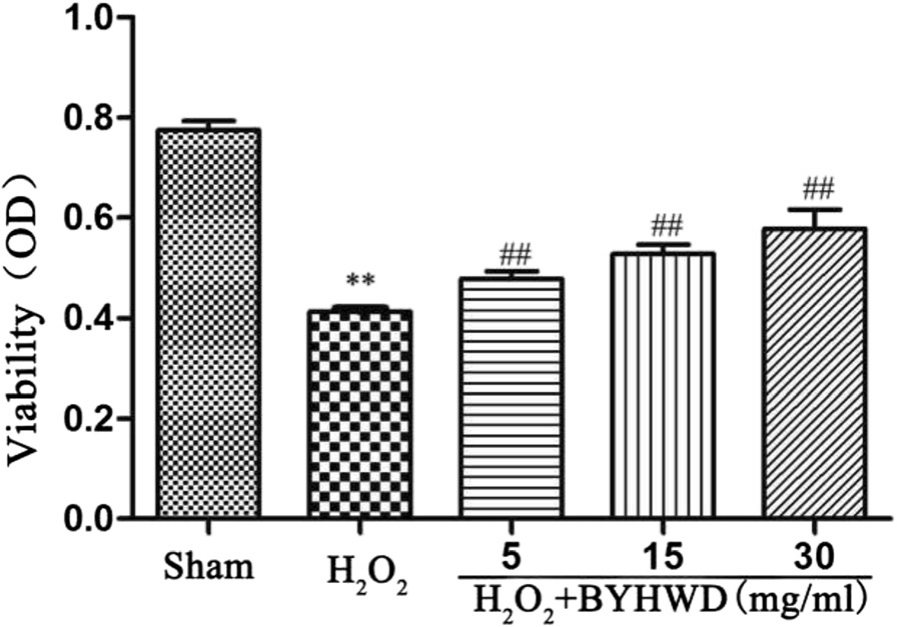
Protective effects of BYHWD on H_2_O_2_-induced viability loss in HUVECs. Quantitative analysis of the effect of BYHWD on H_2_O_2_-induced viability loss in HUVECs. Values are shown as means ± SD (*n* = 6).^**^ *P* < 0.01, compared to sham group. ^##^ *P* < 0.01, compared to H_2_O_2_ group (320 μmol/l)

To evaluate the effect of BYHWD on H_2_O_2_-induced cytotoxicity in HUVECs, we measured the viability of BYHWD-treated HUVECs by MTT assay. BYHWD significantly increased cell viability in a dose-dependent manner after H_2_O_2_-induced cytotoxicity (Fig. 3). These results suggest that BYHWD protects HUVECs from H_2_O_2_-induced injury.

### BYHWD inhibits H_2_O_2_-induced apoptosis in HUVECs

**Fig. 4:**
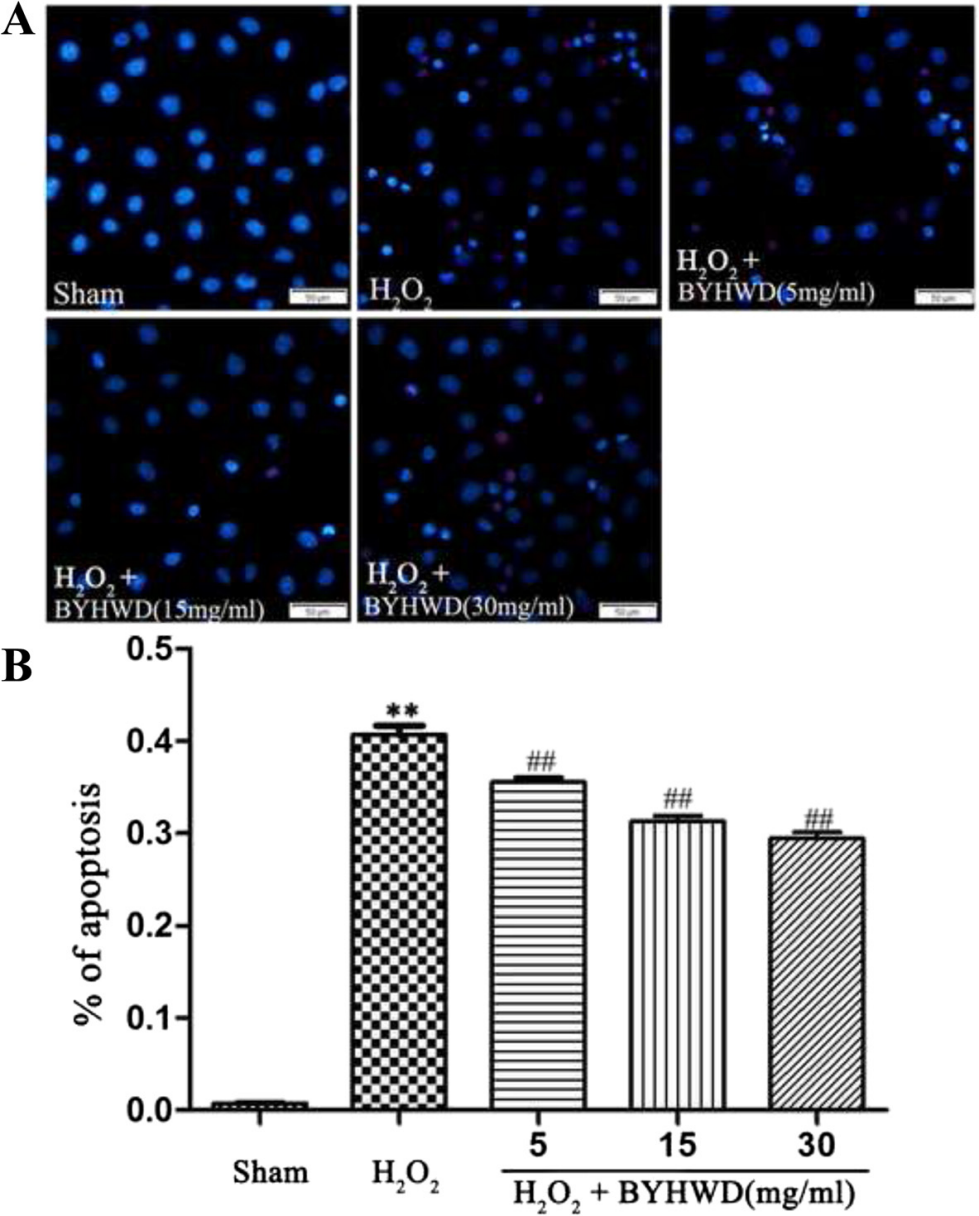
Effects of BYHWD on H_2_O_2_-induced apoptosis in HUVECs. **a** Apoptotic cells were observed using a fluorescence microscope by Hoechst 33342 and PI staining in HUVECs. **b** Quantitative data showed the percentage of apoptotic cells according to treatment in HUVECs. Values are shown as means ± SD (*n* = 10). ^**^ *P* < 0.01, compared to sham group. ^##^ *P* < 0.01, compared to H_2_O_2_ group (320 μmol/l)

To evaluate the effect of BYHWD on H_2_O_2_-induced apoptosis, cells were stained with Hoechst 33342 and PI. Cell nuclei condensed into a bright blue was defined as strongly expressing Hoechst 33342, nornal nuclei with common blue defined as expressing Hoechst 33342. The changes in cell nuclei of apoptotic cells were defined by Hoechst 33342 staining as described previously [16]. Cells expressing Hoechst 33342 and not expressing PI were considered unstressed. Cells strongly expressing Hoechst 33342 and not expressing PI were considered early apoptotic cells. Cells strongly expressing Hoechst 33342 and expressing PI were classified as late apoptotic cells. Cells expressing Hoechst 33342 and strongly expressing PI were considered dead. Exposure to H_2_O_2_ increased apoptosis (Fig. 4) and treatment with BYHWD significantly reduced apoptosis in a dose-dependent manner (Fig. 4). This suggests that BYHWD may block an apoptotic pathway.

### BYHWD decreases H_2_O_2_-induced caspase-3 expression

**Fig. 5:**
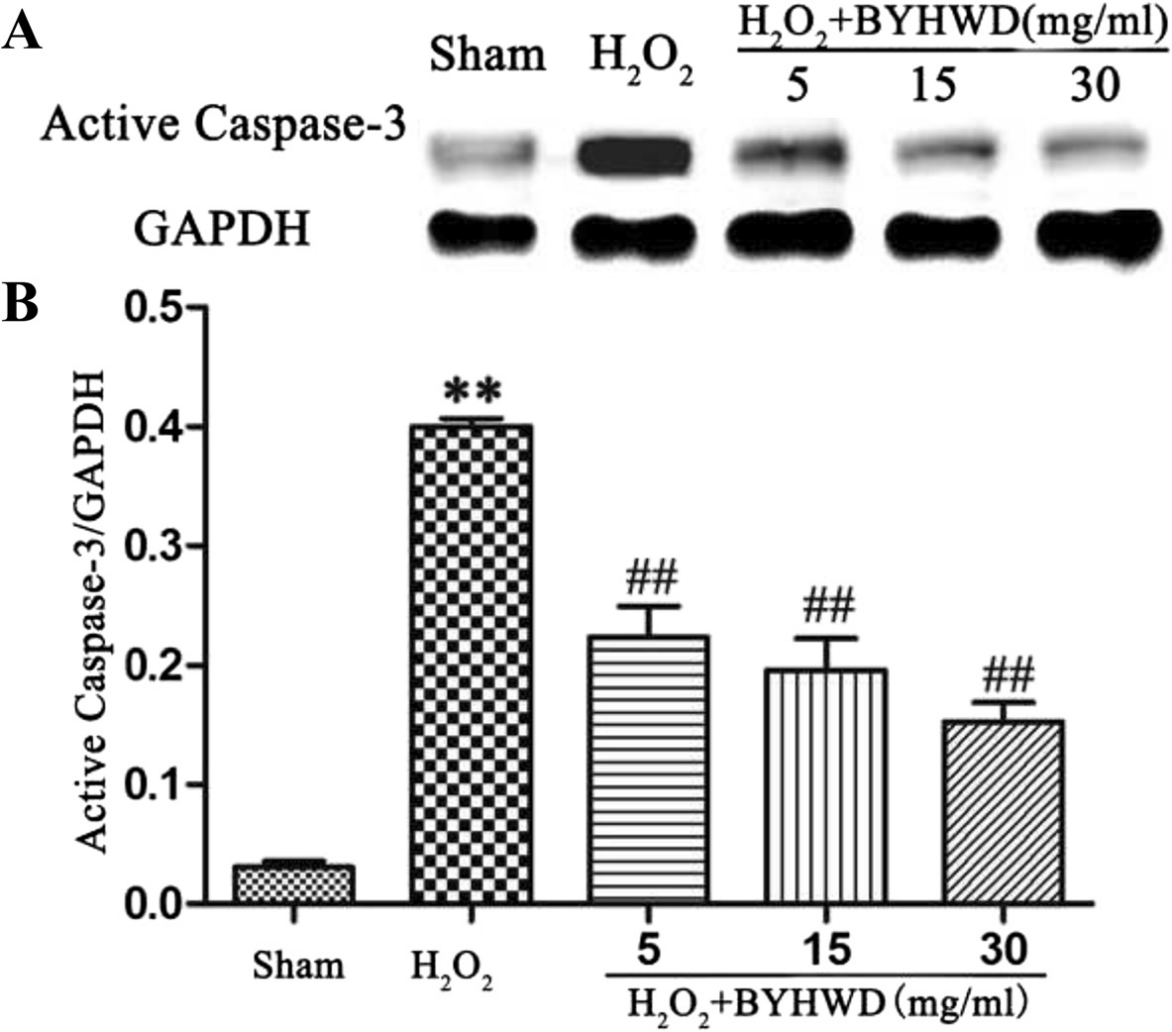
Effects of BYHWD on H_2_O_2_-induced caspase-3 expression in HUVECs. **a** Caspase-3 protein expression in HUVECs were examined by western blotting. **b** Quantitative analysis of band densities for caspase-3 expression in HUVECs were measured. Values are shown as means ± SD (*n* = 3). ^**^ *P* < 0.01, compared to sham group. ^##^ *P* < 0.01, compared to H_2_O_2_ group (320 μmol/l)

H_2_O_2_-induced caspase-3 cleavage is known to regulate cell apoptosis [6]. To determine whether BYHWD affects this process, we measured caspase-3 expression by western blot. Exposure to H_2_O_2_ significantly increased caspase-3 expression (Fig. 5) and BYHWD treatment significantly reduced caspase-3 expression in a dose-dependent manner. These results further verify that BYHWD inhibits H_2_O_2_-induced apoptosis.

### BYHWD inhibits H_2_O_2_-induced ROS production

**Fig. 6:**
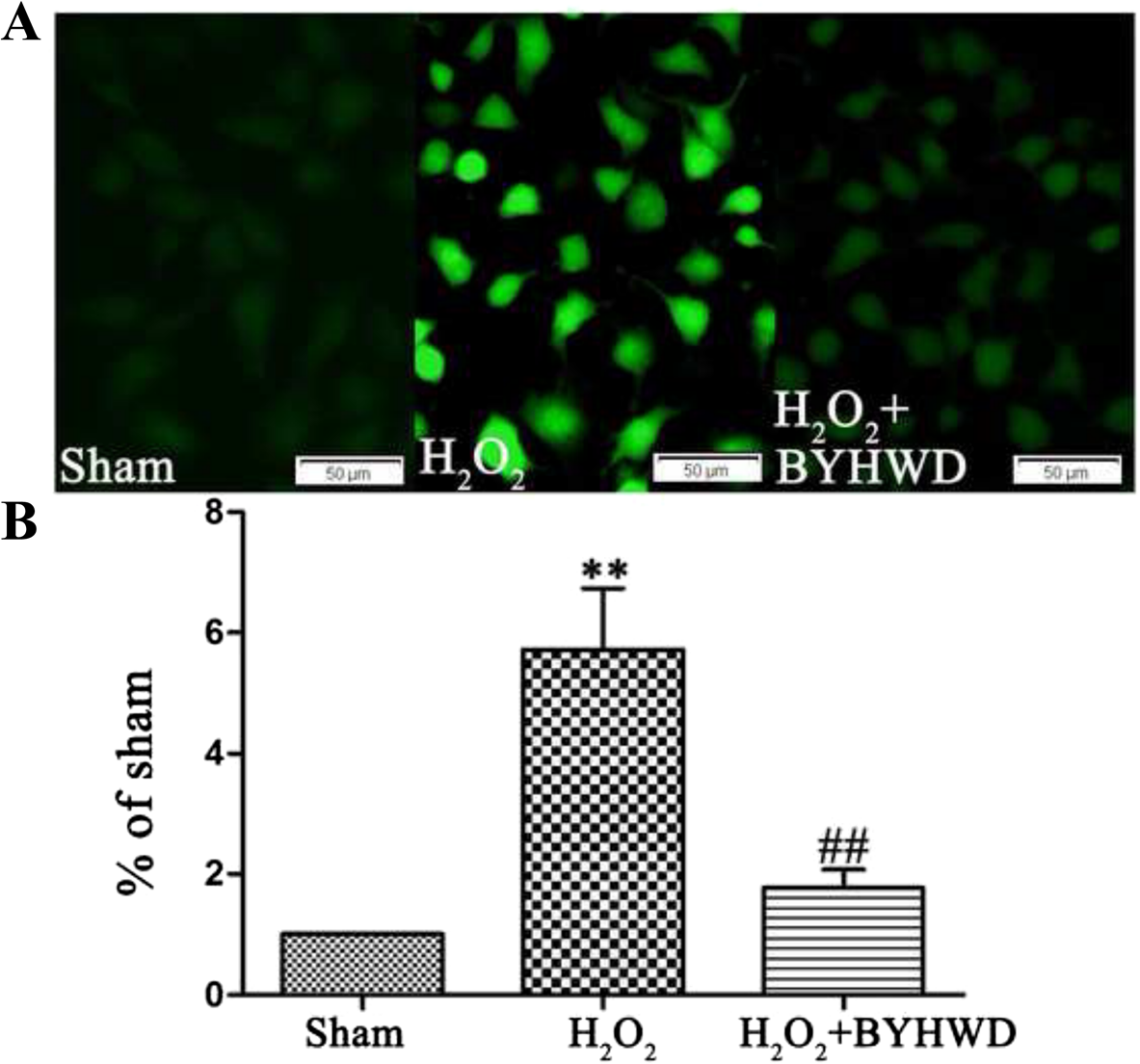
Effects of BYHWD on H_2_O_2_-induced intracellular ROS levels in HUVECs. **a** Representative images of intracellular ROS in HUVECs by fluorescence microscopy.**b** Quantitative analysis of intracellular ROS in HUVECs by spectrofluorophotometry. Values are shown as means ± SD (*n* = 10). ^**^ *P* < 0.01, compared to sham group. ^##^ *P* < 0.01, compared to H_2_O_2_ group (320 μmol/l)

Exogenous addition of H_2_O_2_ induces intracellular ROS production, which affects cell metabolism [17]. To investigate whether inhibition of apoptosis by BYHWD is related to the intracellular production of ROS, we measured intracellular ROS levels using fluorescence microscopy and spectrofluorophotometry with DCFH-DA. DCFH-DA is cleaved intracellularly by esterases and subsequently oxidized to DCFH by ROS. Green fluorescence produced by DCFH indicates abnormally high ROS levels. Exposure to H_2_O_2_ (320 μmol/l) induced a burst of green fluorescence in HUVECs (Fig. 6), which was inhibited by BYHWD. These findings suggest that BYHWD inhibits H_2_O_2_-induced ROS production.

### BYHWD decreased MDA levels and increased SOD levels induced by H_2_O_2_

**Fig. 7:**
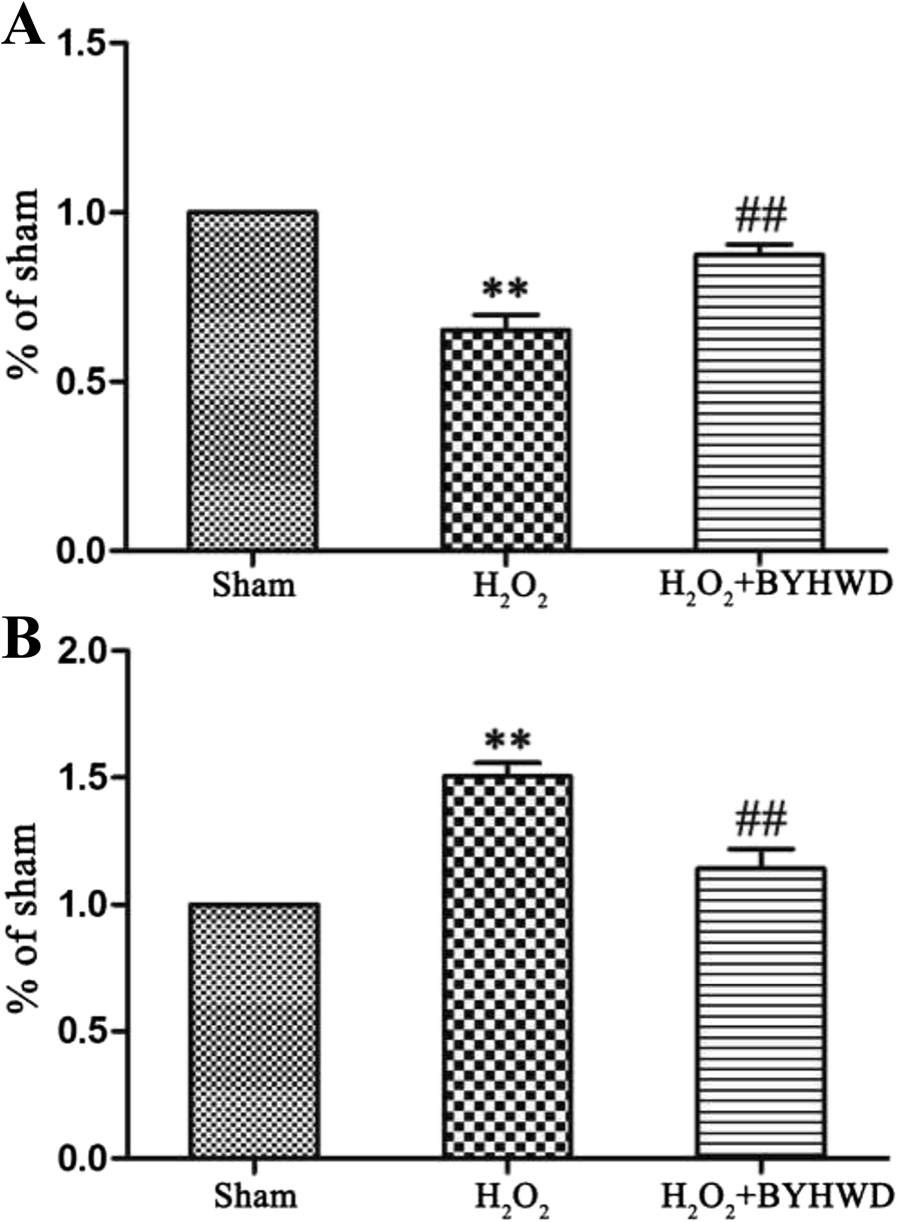
Effects of BYHWD on H_2_O_2_-induced MDA and SOD levels in HUVECs. **a** Quantitative analysis of intracellular MDA levels in HUVECs. **b** Quantitative analysis of intracellular SOD levels in HUVECs. Values are shown as means ± SD (*n* = 10). ^**^ *P* < 0.01, compared to sham group. ^##^ *P* < 0.01, compared to H_2_O_2_ group (320 μmol/l)

MDA levels reflect the degree of lipid peroxidation by ROS-induced cell damage and SOD activity indirectly reflects the ability to eliminate ROS [18]. To determine whether BYHWD affects ROS-induced cell damage, we examined intracellular MDA and SOD levels. Exposure to H_2_O_2_ reduced MDA and increased SOD levels and treatment with BYHWD reduced MDA and further increased SOD levels (Fig. 7).

### BYHWD inhibits H_2_O_2_-induced ultrastructural disruption of mitochondria

**Fig. 8:**
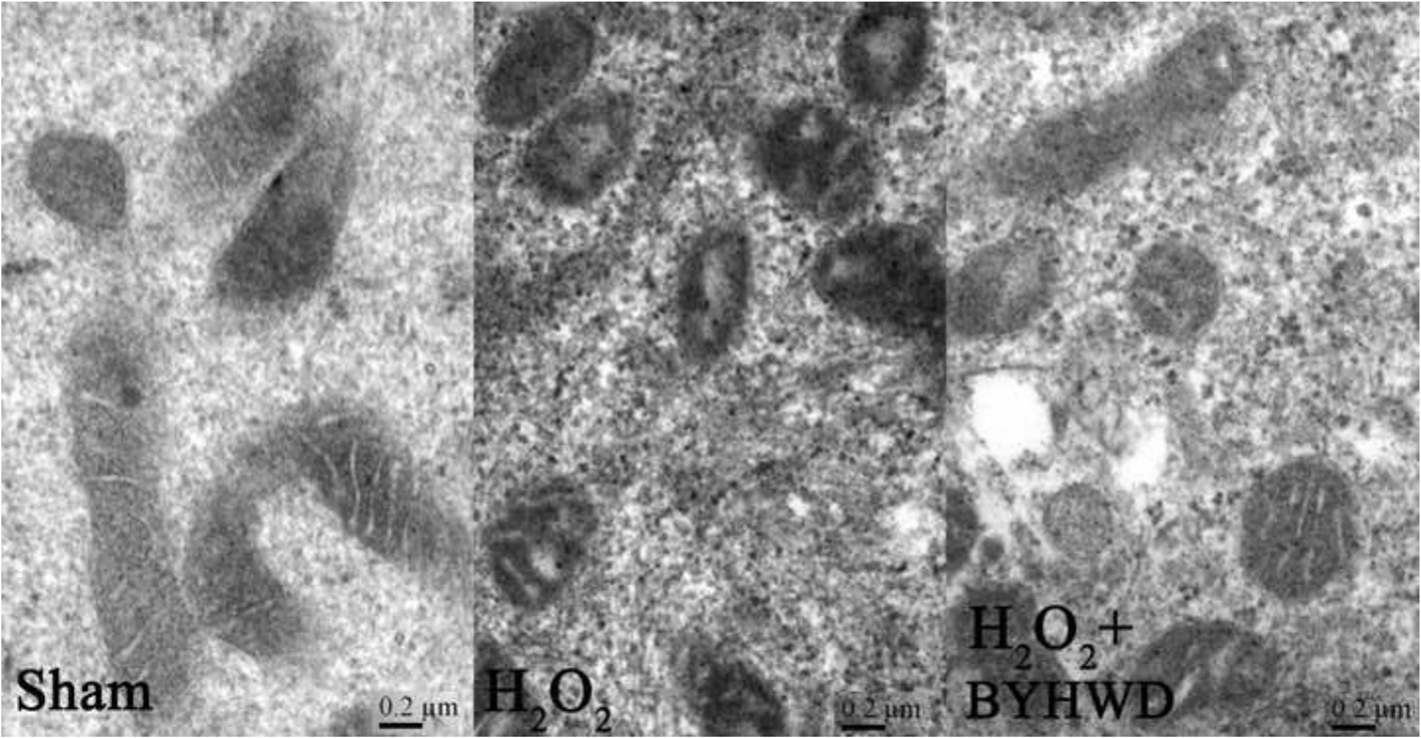
BYHWD inhibits H_2_O_2_-induced ultrastructural disruption of mitochondria in HUVECs. Transmission electron micrographs showed swelling mitochondria, disrupted the mitochondrial membrane, and fragmented the mitochondrial cristae induced by H_2_O_2_ (320 μmol/l) (a and b). Mitochondrial disruption was rescued by BYHWD treatment (30 mg/ml) (c)

Mitochondria are important organelles for cellular energy production and are involved in cell apoptosis [19]. To demonstrate whether inhibition of apoptosis by BYHWD is related to changes in mitochondria ultrastructure, we examined HUVECs by transmission electron microscopy. H_2_O_2_ caused mitochondrial swelling, disrupted the mitochondrial membrane, and fragmented the mitochondrial cristae in HUVECs (Fig. 8). Mitochondrial disruption by H_2_O_2_ was rescued in HUVECs by BYHWD treatment (30 mg/ml). Therefore, BYHWD inhibits H_2_O_2_-induced ultrastructural disruption of HUVEC mitochondria.

### BYHWD restores H_2_O_2_-induced reduction of MMP

**Fig. 9:**
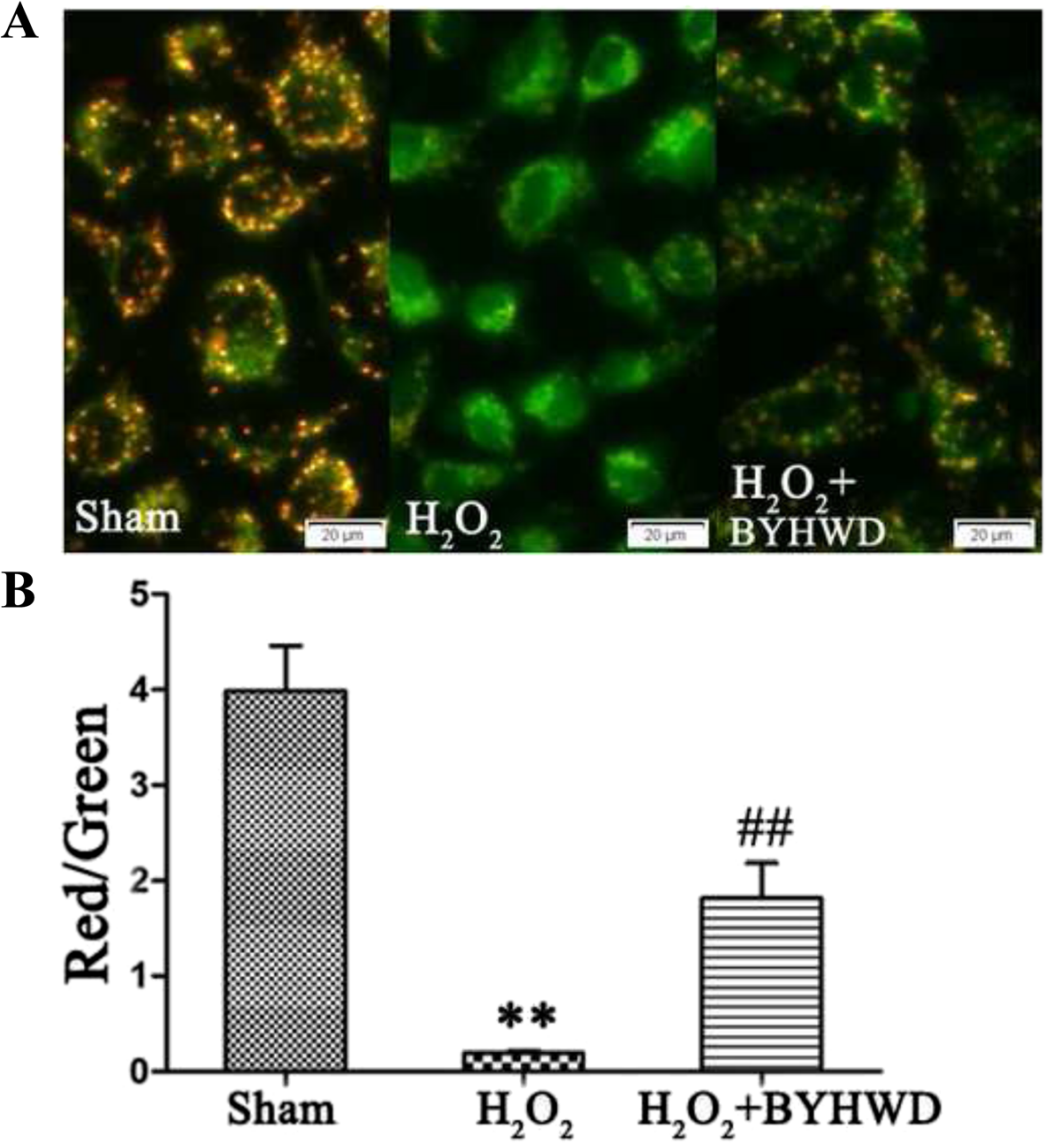
BYHWD restores H_2_O_2_-induced reduction of MMP in HUVECs. **a** Representative images of MMP in HUVECs measured by JC-1. **b** Quantitative analysis of MMP in HUVECs. Values are shown as means ± SD (*n* = 9). ^**^ *P* < 0.01, compared to sham group. ^##^ *P* < 0.01, compared to H_2_O_2_ group (320 μmol/l)

Opening of mitochondrial permeability transition pores by ROS causes mitochondrial dysfunction because of mitochondrial matrix swelling and outer membrane rupture [20]. To examine this, we measured MMP in HUVECs using JC-1. High MMP is indicated by the production of red fluorescence and depolarized regions are indicated by green fluorescence. Exposure to H_2_O_2_ reduced the production red fluorescence and increased the intensity of green fluorescence (Fig. 9). BYHWD treatment increased the production of red fluorescence and reduced the production of green fluorescence, demonstrating that BYHWD can restore H_2_O_2_-induced loss of MPP.

## Discussion

Vascular endothelial cells maintain vascular permeability and vascular responses to a variety of physiological and pathological stimuli [21]. When the structure and function of vascular endothelial cells are impaired, lesions form in the lumen (e.g. thrombosis, atherosclerosis and vasculitis), causing vascular stenosis or occlusion [20]. If this occurs in cerebral vessels, it can cause ischemic cerebrovascular disease, such as transient ischemic attack and cerebral infarction [22]. Endothelial cell proliferation and angiogenesis are important for neurological recovery after cerebral infarction [23]. However, excessive ROS production aggravates endothelial cell injury during cerebral I/R [24, 25]; endothelial cells produce many ROS, which reduces antioxidant activity and sensitivity to exogenous ROS [26]. Neurons also produce ROS, which increases the damage done to endothelial cells by oxygen-free radicals [27]. Preliminary studies have shown that ROS-induced oxidative stress induces lipid peroxidation and protein damage in membranes, alters the antioxidant system, mutates DNA, alters gene expression, and induces vascular endothelial cell apoptosis, thereby aggravating brain ischemia and hypoxia [28]. Protecting vascular endothelial cells from ROS-induced apoptosis may help the treatment of cerebrovascular diseases. HUVECs are the most commonly used model in cardio-cerebrovascular disease research [20, 29]. For this reason, we selected H_2_O_2_-induced apoptosis in HUVECs as an in vitro model of endothelial cell oxidative stress damage after cerebral ischemia.

BYHWD is one of the most classical medicinal prescriptions, comprised of seven active ingredients: Radix Astragali, Radix Angelicae Sinensis, Radix Paeoniae Rubra, Rhizoma Ligustici Chuanxiong, Semen Persicae, Flos Carthami, and Pheretima [13]. Based on the theories of traditional Chinese medicine, the combined effects of multiple herbs within a formula may have a powerfully curative effect. A traditional herbal formulation such as BYHWD is prescribed according to the principles of monarch, minister, assistant and guide. The monarch Radix Astragali is the chief drug for treating the disease, the minister Radix Angelicae Sinensis intensifies the effect of the monarch drug, the assistants Radix Paeoniae Rubra, Rhizoma Chuanxiong, Semen Persicae, and Flos Carthami treat the secondary symptoms or inhibit the toxicity of the monarch drug, and the guide drug Pheretima guides the herbs to the diseased regions and balances the effects of all herbs. Chemical investigations have revealed that the active constituents of BYHWD are numerous and diverse. Radix Astragali contains flavonoids (formononetin, ononin, calycosin and its glycoside), saponins (astragaloside I, II, III, IV, V, VI, VII, VIII), polysaccharides and amino acid; Radix Angelicae Sinensis contains ferulic acid; Radix Paeoniae Rubra contains paeoniflorin, oxypaeoniflorin, benzoylpaeoniflorin and ligustilide; Rhizoma Ligustici Chuanxiong contains tetramethylpyrazine, perlolyrine, ligustilide, ferulic acid, and protocatechuic acid; Semen Persicae contains amygdalin, prunasin, sterol, and organic acid; Flos Carthami contains hydroxysafflor yellow A; and Pheretima contains hypoxanthine [15, 30]. Pharmacological studies also have revealed that the active components of BYHWD contains calycosin-O-β-D-glucoside, formononetin, ononin, calycosin, astragaloside IV and astragaloside I from Radix Astragali, paeoniflorin from Radix Paeoniae Rubra, tetramethylpyrazine, ferulic acid and ligustilide from Radix Angelicae Sinensis and Rhizoma Ligustici Chuanxiong, amygdalin from Semen Persicae, hydroxysafflor yellow A and kaempferol from Flos Carthami [30–32].

BYHWD has been used to treat stroke and stroke-induced disability in China for centuries [33]. Recent studies have reported that BYHWD has a protective effect on nerve cells in humans and animal models [12, 34]. BYHWD exerts its neuroprotective effects by promoting the growth and differentiation of nerve cells [20, 35], and inhibiting neural apoptosis and inflammation after cerebral infarction [36]. However, the protective mechanisms of BYHWD in endothelial cells after brain ischemia are currently undefined. To address this, we explored the protective effects and mechanisms of BYHWD on H_2_O_2_-induced apoptosis in HUVECs.

H_2_O_2_ exposure induced apoptosis, cytoactivity, ROS increase, and mitochondrial disorder in HUVECs. BYHWD inhibited H_2_O_2_-induced apoptosis in HUVECs by reducing intracellular levels of ROS and MDA. In addition, BYHWD increased intracellular SOD levels and restored the MMP and ultrastructural integrity of mitochondria. We have demonstrated for the first time that BYHWD protects against H_2_O_2_-induced apoptosis in HUVECs by inhibiting a ROS-mediated mitochondrial dysfunction pathway. These findings may help to identify novel drug targets and therapies for cerebrovascular diseases.

Apoptosis is a process of programmed cell death in which defective and harmful cells are eliminated to maintain homeostasis [37]. There are three pathways involved in apoptosis: the mitochondrial pathway, death receptor pathway, and endoplasmic reticulum stress pathway [38]. Mitochondria are key regulators of cellular energy homeostasis and play a central role in apoptosis [39]. Mitochondrial apoptotic signals decrease the MMP, causing Cyt-c and Smac and other contents to leak from the mitochondria [4, 5]. Smac speeds up apoptosis and Cyt-c promotes apoptosis by activating caspase-3 [4, 6]. We have shown that H_2_O_2_ causes mitochondrial swelling, disruption of mitochondrial cristae, reduced MMP and activation of caspase-3 in HUVECs, eventually leading to apoptosis. BYHWD treatment reversed these effects and attenuated H_2_O_2_-induced apoptosis through the mitochondrial pathway.

ROS are powerful oxidizing agents in cells and established regulators of apoptosis [40]. Oxidative stress and mitochondrial function are positively correlated. Oxidative stress can cause cellular apoptosis by mitochondria-dependent pathways [41]. H_2_O_2_ can cause endothelial cell injury by inducing mitochondrial dysfunction, including loss of MMP and the MMP transition, which increases the formation of ROS by inhibiting the respiratory chain [20, 29]. Excessive ROS cause peroxidation of polyunsaturated fatty acids and lipids, damaging membrane fluidity, permeability and integrity. MDA levels reflect the degree of lipid peroxidation and indirectly the degree of cell damage. SOD is a free-radical scavenger and SOD levels indirectly reflect the level of ROS elimination [18]. We observed that intracellular ROS and MDA levels increased and SOD levels decreased in HUVECs exposed to H_2_O_2._ BYHWD reduced ROS production in HUVECs, which increased the activity of SOD and reduced MDA levels, indicating that BYHWD has antioxidant effects. We propose that these effects represent the mechanism by which BYHWD inhibits H_2_O_2_-mediated apoptosis in HUVECs.

## Conclusion

We have shown that BYHWD significantly attenuates H_2_O_2_-induced apoptosis in HUVECs. Furthermore, BYHWD inhibited intracellular ROS production, increased SOD activity, reduced MDA levels, reversed MMP decline and rescued structural disruption of mitochondria. These findings suggest that the anti-apoptotic mechanisms of BYHWD involve inhibiting ROS-mediated mitochondrial dysfunction pathways in HUVECs.

## Abbreviations

BYHWD, Buyang Huanwu Decoction; HUVECs, Human umbilical vein endothelial cells; ROS, Reactive oxygen species; MMP, Mitochondrial membrane potential; (I/R), Cerebral ischemia/reperfusion; SOD, superoxide dismutase; MDA, Malondialdehyde; PI, Propidium iodide.
